# Trip East

**Published:** 1863-07

**Authors:** 


### THE TRIP EAST.

Arrangements have been made for excursion tickets from here to Philadelphia, and we presume will be from there to Saratoga; this will be at one half the usual rates. Those going from here will start on the 26th on the 10, 40 P. M. train.

7 o'clock A. M. of the 27th will reach Philadelphia on Tuesday at 10 A. M. but as that is the hour for the meeting, a little earlier arrival would be desirable. The 10, 40 train arrives there at 6 o'clock on Tuesday morning. T.

